# Out-of-pocket healthcare payments on chronic conditions impoverish urban poor in Bangalore, India

**DOI:** 10.1186/1471-2458-12-990

**Published:** 2012-11-16

**Authors:** Upendra Bhojani, BS Thriveni, Roopa Devadasan, CM Munegowda, Narayanan Devadasan, Patrick Kolsteren, Bart Criel

**Affiliations:** 1 Institute of Public Health, 250, 2 C Cross, 2 C Main, Girinagar, First Phase, Bangalore 560085, Karnataka, India; 2 Department of Public Health, Institute of Tropical Medicine, Nationalestraat 155, 2000 Antwerp, Belgium

**Keywords:** Chronic, Impoverishment, Healthcare expenditure, Out-of-pocket, India, Outpatient care

## Abstract

**Background:**

The burden of chronic conditions is on the rise in India, necessitating long-term support from healthcare services. Healthcare, in India, is primarily financed through out-of-pocket payments by households. Considering scarce evidence available from India, our study investigates whether and how out-of-pocket payments for outpatient care affect individuals with chronic conditions.

**Methods:**

A large census covering 9299 households was conducted in Bangalore, India. Of these, 3202 households that reported presence of chronic condition were further analysed. Data was collected using a structured household-level questionnaire. Out-of-pocket payments, catastrophic healthcare expenditure, and the resultant impoverishment were measured using a standard technique.

**Results:**

The response rate for the census was 98.5%. Overall, 69.6% (95%CI=68.0-71.2) of households made out-of-pocket payments for outpatient care spending a median of 3.2% (95%CI=3.0-3.4) of their total income. Overall, 16% (95%CI=14.8-17.3) of households suffered financial catastrophe by spending more than 10% of household income on outpatient care. Occurrence and intensity of financial catastrophe were inequitably high among poor. Low household income, use of referral hospitals as place for consultation, and small household size were associated with a greater likelihood of incurring financial catastrophe.

The out-of-pocket spending on chronic conditions doubled the number of people living below the poverty line in one month, with further deepening of their poverty. In order to cope, households borrowed money (4.2% instances), and sold or mortgaged their assets (0.4% instances).

**Conclusions:**

This study provides evidence from India that the out-of-pocket payment for chronic conditions, even for outpatient care, pushes people into poverty. Our findings suggest that improving availability of affordable medications and diagnostics for chronic conditions, as well as strengthening the gate keeping function of the primary care services are important measures to enhance financial protection for urban poor. Our findings call for inclusion of outpatient care for chronic conditions in existing government-initiated health insurance schemes.

## Background

With an epidemiological transition underway in India, the burden of chronic and non-communicable diseases is on the rise. In 2005, these conditions were responsible for 53% of all deaths, and their proportional impact is expected to increase to 67% in 2020 [1]. Chronic conditions, which include most non-communicable diseases but also some communicable diseases, require continuous medical care complemented by long-term support from healthcare services. Responding to the care demands of people with chronic conditions is a challenge in most low- and middle-income countries, including India, where the health system is weak and remains primarily oriented towards the management of infectious diseases, and maternal and child healthcare [1-3]. Health system strengthening is increasingly advocated as a central strategy in the endeavour to improve care for chronic conditions in these countries [2,4].

Health financing is one of the building blocks of health systems [5,6]. Health systems, ideally, should be financed in a way that people can use healthcare services without financial hardship [5]. In India, 71.1% of healthcare is financed through out-of-pocket (OOP) payments by households at the time and point of healthcare use [7]. In 2004–2005, 64.4% of households in India had to incur OOP payments for healthcare [8]. OOP payments act as the primary barrier to access healthcare services in India, and lead to significant impoverishment among those who use the services [8,9]. In fact, Berman and colleagues [10] reported that in 2004, approximately 6.2% of Indians fell below the poverty line due to OOP payments for healthcare; a greater proportion of them for outpatient care (4.9%) than for inpatient care (1.3%), while expenditure for medications constituted the greatest share (71.2%). We hypothesise that people with chronic conditions are likely to incur higher OOP payments for outpatient care, as they need periodic outpatient visits and regular medication on a long-term basis. Such payments may impoverish them and even push them below the poverty line.

Despite several recent studies examining OOP payments for healthcare in India, very few of them report findings disaggregated by type of care (outpatient/inpatient), location (urban/rural) and type of ailments (acute/chronic). Only one study from West Bengal reported some findings on OOP payments for chronic disease care, and its impact on households [11,12]. This study showed that households spent 4.1% of their annual expenditure on chronic disease care. It also indicated that the OOP payments for outpatient care were more strongly associated with financial catastrophe than those for inpatient care.

The purpose of this study is to contribute to this knowledge gap by investigating whether and how OOP payments for outpatient care affect individuals with chronic conditions in Kadugondanahalli (KG Halli), a poor urban neighbourhood in South India.

### Context

KG Halli, a site of this study, is one of the 198 administrative units of Bangalore city, a metropolitan capital of Karnataka. KG Halli has a population of more than 44,500 individuals spread over 0.7 square kilometre [13]. KG Halli includes an area classified as a slum, and the median income of KG Halli residents is INR 73.3 (USD 1.5) per capita per day [13]. A ‘slum’ is a compact settlement of poorly built tenements with inadequate sanitary and drinking water facilities [14]. The population in KG Halli is a social mix, with people speaking five different languages and representing all major religions of the country.

KG Halli also has pluralistic healthcare services. Government provides care through an urban health centre and a community health centre, run by the municipal and provincial governments, respectively. These facilities provide outpatient care and outreach services using allopathic (or ‘modern’) medicine. These health centres provide free care to people living below the poverty line, whereas other users need to pay nominal user fees for some of the services. Additionally, there are at least 32 private healthcare providers (excluding dentists and paramedics) from various systems of medicine (primarily Unani, Ayurveda, Allopathy and Homeopathy) [13]. Private healthcare provision is through several single-doctor clinics and four private hospitals. All of the private facilities provide outpatient care, but only the hospitals provide inpatient facilities, with their capacity ranging from 50 to 100 beds. There are several private pharmacies and laboratories in KG Halli. Private sector is largely unregulated and works on fee-for-service basis [13].

KG Halli is the field site for the Urban Health Action Research Project (UHARP) of the Institute of Public Health (IPH), Bangalore, since 2009 [15]. KG Halli was purposefully selected for the UHARP to study how access to quality healthcare could be improved in a poor urban community with a pluralistic healthcare system. The residents as well as healthcare providers in KG Halli have identified difficulties in affording healthcare as one of the major issues in the area.

The specific objectives of the present study were therefore to assess the i) incidence and extent of the OOP payments on outpatient care for chronic conditions; ii) incidence of the financial catastrophe due to OOP payments; and iii) resultant impoverishment among residents of KG Halli. The result of this study would feed into UHARP and serve as an avenue for discussion and action by stakeholders in the area to improve affordability of chronic condition care.

## Methods

### Study design

We report findings from a large cross-sectional study (census) conducted between June 2009 and March 2010 covering all of the households in KG Halli. Of the 9299 households covered in the census, we further analysed data of 3202 households that reported having one or more members with a chronic condition.

### Data collection and management

We collected data using a structured household-level questionnaire with close-ended questions about socio-demographic characteristics, self-reported illness profile, healthcare seeking behaviour, and healthcare expenditure. The questionnaire was developed in English and translated into *Kannada*, a regional language commonly spoken in KG Halli.

**Figure 1:**
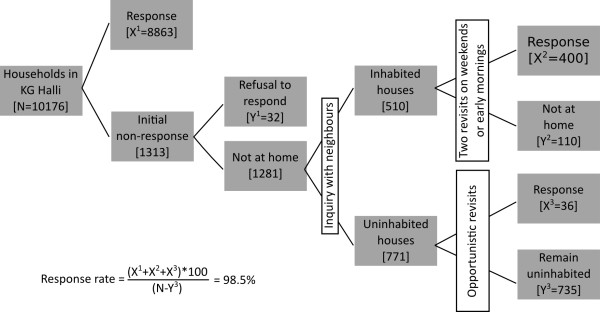
**Sample constitution.** This figure depicts the number of households included in the survey at various stages. Apart from the specific strategies used to enhance the response rate, this figure explains how the response rate (98.5%) to the survey was calculated.

The questionnaire was field tested and refined. Trained data collectors, who were from a similar socioeconomic background to that of the respondents and were fluent in regional languages, administered the questionnaire. Given that most earning members in the households go out for work for most of the day, the only eligibility criterion we employed for choosing the respondent was to have any family member present aged 18 years or older. The flowchart in Figure 1 reveals strategies employed to improve the response rate and the households included at various stages of the census.

All the completed questionnaires were examined for internal validation. Data were externally validated by randomly selecting one in twenty completed questionnaires and revisiting the household to confirm responses. Trained data entry operators entered the data using EpiData Entry 3.1 (The EpiData Association, Odense Denmark).

### Ethics considerations

At the time of this study, the Institute of Public Health, Bangalore did not have Institutional Ethics Committee, and a policy requiring a formal ethics approval for non-clinical survey research. However, we followed ethical principles set for such research.

Considering the low literacy level, linguistic pluralism, and perceived worries/reservations around signing documents among sample population, we preferred to seek informed verbal consent from participants. The participants were explained, in the language that they were confortable with, the purpose of the study, voluntary and anonymous nature of participation in the study, including their rights to withdraw participation at any stage during questionnaire administration. Outcome of the consent process was recorded (as refusal or agreement) in the questionnaire. Data on refusal by participants was maintained (Figure 1). Confidentiality of participants and their family members was assured. Data privacy was maintained: physical forms were stored in a locked metal container at the Institute of Public Health, whereas electronic data were stored in a secured folder in computers of first and second authors. Only researchers of UHARP had access to the data, only for the research purpose.

### Measures and analyses

The dependent variables used in this study are described below.

#### OOP payments for healthcare

We measured OOP payment as the sum of all healthcare related expenditures made by individuals/households within 30 days preceding the census at the time when healthcare services were received [16]. We collected data on ‘direct medical care’ (i.e., expenditures for consultation fees, facility charges, expenses for medications and laboratory investigations) and ‘other’ indirect expenditures (i.e., expenditures for travel, food, and any informal payments, such as bribes or kickbacks). We report the incidence of OOP payments and the median OOP payment per month.

#### Catastrophic healthcare expenditure (CHE)

We measured CHE and its impact on households/individuals using the technique and indicators adapted from O’Donnell et al. [17]. We measured the incidence, intensity, and distributional fairness (across income quintiles) of CHE. We used household monthly income as a denominator in calculating the CHE instead of the usually recommended household consumption expenditure, or non-food expenditure, because we did not have data on the latter.

#### Headcount

Headcount is the percentage of households whose monthly OOP expenditure, as fraction of monthly household income, for outpatient care (for chronic conditions) exceeded a particular threshold. Most commonly accepted and used threshold in literature has been 10% at which households are usually forced to cut down their subsistence needs [18]. We calculated the headcount at four different thresholds i.e. 5%, 10%, 15%, and 20% using the following formula,

$$\text{Headcount}=\left(\frac{1}{N}\right)\overset{N}{\underset{i=1}{\sum }}E_{i}$$

[17] where *E* is an indicator equal to one if *T*_*i*_/*X*_*i*_ > *z* and zero otherwise, *T*_*i*_ is the OOP expenditure by a household *i*, *X*_*i*_ is the income of a household *i*, *z* is the given catastrophic threshold, and *N* is the sample size.

#### Overshoot

Headcount only suggests the percentage of households that spent OOP beyond a particular threshold but does not give an idea on how far (intensity) they spent beyond the threshold. Overshoot measures the degree by which an average OOP expenditure (in entire sample) crossed the given catastrophic threshold. We measured the overshoot by using the following formula,

$$\text{Overshoot}=\left(\frac{1}{N}\right)\overset{N}{\underset{i=1}{\sum }}O_{i}$$

[17] where the excess payment of household *i* is defined as *O*_*i*_ = *E*_*i*_((*T*_*i*_/*X*_*i*_) − *z*).

#### Mean positive overshoot

Unlike the overshoot that uses all the households as denominator, the Mean Positive Overshoot (MPO) uses only those households that have actually experienced CHE as the denominator. Hence MPO measures the degree by which the average OOP expenditure by households that have experienced catastrophe has exceeded the given catastrophic threshold. We measured the MPO by using the following formula,

$$\text{Mean}\;\text{positive}\;\text{overshoot}\left(MPO\right)=\text{Overshoot}/\text{Headcount}$$

[17]. Hence if household *i* experienced the CHE, it would have spent (*MPO*_*i*_ + *z*) percentage of the household income on healthcare.

#### Concentration curve & index

Concentration curve and index help to understand the distribution of CHE across the income quintiles. Concentration curve above the 45-degree line (line of equality) leads to negative value for concentration index and suggests disproportionately higher concentration of catastrophe among the poor households and vice versa. When the concentration index is zero, it suggests the absence of the income-related inequalities in distribution of CHE. Concentration index has been calculated using the following formula,

$$\begin{array}{l} \text{Concentration}\;\text{index}\;\left(CI\right)=\left(p1L2-p2L1\right)\\ \;+\left(p2L3-p3L2\right)\\ \;+\left(p3L4-p4L2\right)\\ \;+\left(p4L5-p5L4\right) \end{array}$$

[17] where *p* is the cumulative percentage of households ranked by their monthly income, *L* is the cumulative percentage of households experiencing catastrophe for the corresponding *p*. Numbers (1 to 5) suggest the relevant income quintile.

The independent variables used in the study are described below.

#### Chronic condition

There is no standard definition available for a chronic condition. It is generally defined as an illness or impairment that lasts for a long duration. The minimum time period for an illness to be considered chronic varies depending on the source of the definition: ranging from three months to one year [19,20]. We considered the presence of a chronic condition when any individual was taking medications on a daily basis for 30 days preceding the census. Respondents usually reported cases where family members were prescribed regular medication by a healthcare provider but were unable to take the medication for various reasons. We recorded such instances as the presence of chronic conditions.

#### Type of healthcare services – as place for consultation

The type of healthcare service was defined as either government or private, depending on the ownership of the healthcare facility used by patient as the first contact point where consultation happened. This does not necessarily mean that all of the healthcare was received at this facility. For example, a person with diabetes who contacts a government health centre for a consultation might be asked to use a private laboratory for blood sugar measurements and/or a private pharmacy for medications if these services are not available at this facility. This is, unfortunately, often the case. In this example, we would define the government healthcare service as being the place for consultation. The patient’s healthcare expenditure, however, would include the expenditures for services received from the government health centre as well as those incurred for private laboratory and pharmacy services.

#### Levels of healthcare services

We defined three levels of healthcare services, depending on where the person with a chronic condition was being managed at the time of the census: i) clinics/health centres; ii) referral hospitals with in-patient facilities; and iii) super-specialty hospitals attached to the medical schools. These three groups of facilities roughly correspond to primary, secondary and tertiary healthcare services, respectively. However, it is important to note that in India, the levels of healthcare services are not very distinct. In other words, there is poor gate keeping e.g., outpatient care for minor ailments and/or chronic conditions is often provided by all three levels rather than being limited to only clinics/health centres.

We used the per capita household income, household size, and the type of ration card as other independent variables. A ration card is a document issued to households by government authorities to enable access to essential commodities at subsidised rates. It has also become an important identity card for households’ official poverty status (above or below the poverty line) to access many welfare schemes.

We used STATA® 11 (StataCorp LP, Texas, USA) to perform univariate and bivariate analyses. Associations and comparisons between variables were assessed using the chi-squared and Wilcoxon rank-sum test.

## Results

The overall response rate for the census was 98.5% (Figure 1). The median age of the respondents was 35 years, and 75.1% of them were women. Of the 9,299 families surveyed, 3202 families (34.4%) reported having one or more family member (total 3844 individuals) with a chronic condition. Of those who reported presence of chronic condition, 3029 families (94.6%) or 3782 individuals (98.4%) sought healthcare from healthcare facilities at some point in time. Rest of them either did not seek care or used self-medication. Table 1 provides the major characteristics of the sample population.

**Table 1:** Major characteristics of the sample population

**Households (n=9299)**			
		**Households that reported chronic condition (n=3202)**	**Households that did not report chronic condition (n=6097)**
**Income per month** in INR - [Median (range)]	Household Income	12000 (0, 205000)	9000 (14, 195000)
	Per capita income	2500 (0, 60001)	2250 (2.8, 43333.3)
	First quintile	1250 (0, 1583.3)	1200 (2.8, 1480)
	Second quintile	1952.4 (1600, 2181.8)	1600 (1500, 1950)
	Third quintile	2500 (2200, 2925)	2250 (2000, 2657.1)
	Fourth quintile	3333.3 (3000, 3916.7)	3000 (2666.7, 3750)
	Fifth quintile	5000 (4000, 60001)	5000 (3800, 43333.3)
**Poverty status** – as per the ration card [n (percentage)]	Above the poverty line	1972 (61.6)	2683 (44.3)
	Below the poverty line	242 (7.6)	725 (12.0)
	No ration card	988 (30.9)	2643 (43.6)
**Household size** [mean (SD)]		5.2 (2.3)	4.6 (1.8)
**Religion** [n (percentage)]	Islam	2178 (68.3)	3381 (64.2)
	Hinduism	666 (20.9)	1468 (24.3)
	Christianity	352 (11.0)	677 (11.2)
	Others	2 (0.1)	17 (0.3)
**Chronic Conditions (n=3902)**			
**Type of the health services** as place for consultation^*^ [n (percentage)]	Government	742 (19.6)	
	Private	3040 (80.1)	
**Levels of the health services** [n (percentage)]	Clinics/Health centres	1621 (41.5)	
	Hospitals	1466 (37.6)	
	Super-specialty hospitals	695 (17.78)	

^*^Number of ailments treated in government and private sector does not add up to the total (i.e. 3902) because, for 120 ailment instances, individuals either used self-medication or did not seek care.

### OOP payments

We found that 69.6% (95%CI=68.0-71.2) of households made OOP payments for outpatient care for chronic conditions in the 30 days preceding the census. Overall, 68.1% (95%CI=66.6-69.5) of the chronic conditions led to OOP payments. The incidence of OOP payments varied according to the type and level of healthcare services sought (Table 2). There was no statistically significant difference in the incidence of OOP payments between government (72.5%) and private (69.3%) sectors as a place for consultation. The incidence of OOP payments was greatest at the level of super-specialty hospitals, followed by referral hospitals and clinics/health centres. However, this difference was statistically significant only when the government sector was used as a place for consultation. The odds for incurring OOP payments was 2.6 times greater (95%CI=1.7-3.9) for ailments treated at super-specialty government hospitals compared with government health centres.

**Table 2:** Incidence and extent of OOP payments according to type and levels of healthcare services

**Income per capita**	Incidence of OOP payments (Ailment as unit)* (95%CI)						**Median share of household income spent as OOP (%)**	
	Government^**^(n=742)			Private^**^(n=3040)			**Government**	**Private**
	**Clinics/ Health centres (n=186)**	**Referral hospital (n=171)**	**Super-specialty hospital (n=385)**	**Clinics/ Health centres (n=1435)**	**Referral hospital (n=1295)**	**Super-specialty hospital (n=310)**		
1^st^ quintile	56.9 (44.6, 69.3)	72.4 (55.1, 89.7)	77.8 (70.1, 85.4)	68.8 (63.2, 74.5)	69.2 (62.5, 76.0)	75.0 (64.4, 85.6)	4.0	5.9
2^nd^ quintile	54.5 (39.2, 69.9)	78.9 (58.8, 99.1)	79.4 (69.6, 89.3)	61.5 (56.2, 66.9)	72.3 (66.3, 78.2)	78.6 (65.6, 91.5)	2.5	3.9
3^rd^ quintile	60.7 (41.4, 80.0)	62.2 (45.8, 78.6)	81.3 (72.5, 90.0)	68.8 (63.6, 74.1)	66.5 (60.5, 72.6)	62.7 (50.0, 75.4)	2.4	2.9
4^th^ quintile	81.8 (64.3, 99.3)	75.9 (59.3, 92.4)	86.4 (75.8, 96.9)	70.4 (64.2, 76.5)	70.3 (64.5, 76.1)	72.7 (60.6, 84.9)	2.0	3.1
5^th^ quintile	76.6 (56.3, 96.1)	50.0 (36.2, 63.8)	79.9 (69.1, 90.2)	71.8 (66.6, 77.0)	73.8 (69.3, 78.2)	69.2 (58.8, 79.7)	1.3	2.4

*We used ailment as a unit of analysis instead of households. This is because individuals from a single household might seek care from different type (and levels) of health services making it impossible to do segregated analysis as presented in this table. ^**^Number of ailments treated in government and private sector does not add up to the total (i.e. 3902) because, in 120 ailment instances, individuals either used self-medication or did not seek care.

For the households that made OOP payments, the monthly median OOP payment on outpatient care was INR 400 (95%CI=380-403.5) (USD 8.1). The median OOP payments on direct medical care was INR 360 (USD 7.3). Median OOP payments on the other items (indirect expenditure) was zero, meaning 50% of households did not incur OOP payments on such items. The median OOP payment per chronic condition was INR 320 (95%CI=300-350) (USD 6.5), with greatest share on direct medical care.

The median monthly OOP payment per chronic condition was significantly greater when private sector was used as place for consultation (INR 415 or USD 8.4) compared to the government sector (INR 280 or USD 5.7) (See Additional file 1). This finding was primarily due to significantly greater OOP payments for direct medical care when the private sector was used as place for consultation rather than the government sector. Collective OOP payments on other items, including travel, food, and informal payments, were significantly greater when the government sector was used as place for consultation.

The OOP payments increased across the levels of healthcare services when the government sector was used as place for consultation. The median OOP payments made at health centres was significantly lower compared to that made at the referral hospitals or the super-specialty hospitals. Such differences were not significant when the private sector was used as place for consultation.

**Figure 2:**
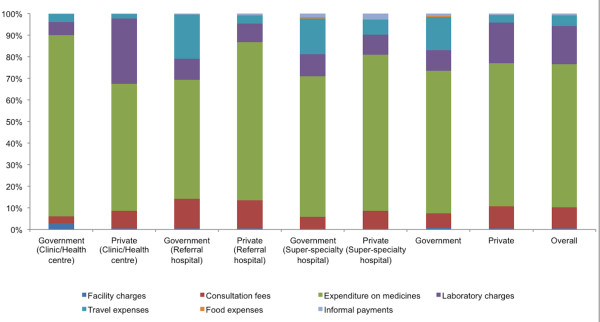
**Composition of OOP payments according to the type and levels of healthcare services.** This figure depicts the composition of out-of-pocket payments for outpatient care for chronic conditions according to the type (i.e., government, private) and the level (i.e., clinics/health centres, referral hospitals, super-specialty hospitals) of healthcare services. The greatest share of out-of-pocket payments (66.3%) was on the purchase of medications irrespective of the type and the level of healthcare services used. Apart from the expenditures on medications, the laboratory investigations and the consultation fees of doctors took the greatest shares of OOP payments at the health centres/clinics and referral hospital levels. At the super-specialty level, expenditures on travel to healthcare facilities became the second largest expenditure.

Irrespective of the type and the level of healthcare services used, households spent the greatest share of OOP payments (66.3%) on the purchase of medications (Figure 2). Apart from the expenditures on medications, the laboratory investigations and the consultation fees of doctors took the greatest shares of OOP payments at the health centres/clinics and referral hospital levels. At the super-specialty level, expenditures on travel to healthcare facilities became the second largest expenditure. The expenditures on travel were greater when the government sector was used as place for consultation, especially at referral hospitals (20.6% of OOP payments) and at super-specialty hospitals (16.4% of OOP payments), making it the second major source of OOP payments at these levels.

Households spent a median of 3.2% (95%CI=3.0-3.4) of their income on OOP payments for outpatient care for chronic conditions. This share was greater when the private sector (3.3%) was used as place for consultation compared with the government sector (2.4%). The difference was statistically insignificant.

OOP payments were regressive. The median share of household income spent on OOP payments was significantly higher among the lowest income quintile compared with the highest income quintile (Table 2).

### Catastrophic healthcare expenditures

The incidence and intensity of CHE across the income groups are provided in Table 3. At any given threshold, the incidence of financial catastrophe (i.e. the ‘Headcount’) was the greatest among the poorest households and decreased with an increase in income, except for the fourth quintile, for which the headcount was slightly higher than that of the third quintile. Concentration of financial catastrophe among the poorest households was also evident form the concentration curves being above the line of equality, and the negative concentration indices for all the four catastrophic thresholds (See Additional file 2).

**Table 3:** Incidence and intensity of CHE across the income groups

**Income groups**	**Measures of CHE**	**Catastrophic threshold (share of household income) used to measure CHE**			
		**5%**	**10%**	**15%**	**20%**
**First quintile (Poorest)**	Headcount (%) (95%CI)	38.1 (34.2, 41.9)	23.1 (19.7, 26.4)	14.2 (11.4, 16.9)	11.0 (8.5, 13.4)
	Overshoot (%) (95%CI)	6.7 (4.5, 8.9)	5.3 (3.2, 7.4)	4.4 (2.4, 6.5)	3.8 (1.8, 5.8)
	Mean Positive Overshoot (%)	17.6	22.9	31.0	34.5
**Second quintile**	Headcount (%) (95%CI)	27.4 (23.9, 30.9)	13.8 (11.1, 16.6)	6.6 (4.6, 8.6)	4.7 (3.0, 6.3)
	Overshoot (%) (95%CI)	2.3 (1.8, 2.9)	1.4 (0.9, 1.8)	0.9 (0.5, 1.3)	0.6 (0.3, 1.0)
	Mean Positive Overshoot (%)	8.4	10.1	13.6	12.8
**Third quintile**	Headcount (%) (95%CI)	20.7 (17.5, 23.8)	10.2 (7.8, 12.5)	6.0 (4.2, 7.9)	4.1 (2.6, 5.7)
	Overshoot (%) (95%CI)	2.1 (1.4, 2.7)	1.4 (0.8, 1.9)	1.0 (0.5, 1.5)	0.7 (0.3, 1.2)
	Mean Positive Overshoot (%)	10.1	13.7	16.7	17.1
**Fourth quintile**	Headcount (%) (95%CI)	23.7 (20.0, 27.4)	11.6 (8.8, 14.4)	7.6 (5.3, 10.0)	4.9 (3.0, 6.8)
	Overshoot (%) (95%CI)	5.6 (−5.7, 11.7)	4.7 (−1.4, 10.8)	4.3 (−1.8, 10.3)	4.0 (−2.1, 10.0)
	Mean Positive Overshoot (%)	23.6	40.5	56.6	81.6
**Fifth quintile (Least poor)**	Headcount (%) (95%CI)	16.9 (14.2, 19.7)	8.2 (6.2, 10.2)	2.2 (1.1, 3.3)	0.3 (−0.1, 0.7)
	Overshoot (%) (95%CI)	1.1 (0.6, 1.5)	0.5 (0.1, 0.9)	0.3 (−0.1, 0.7)	0.2 (−0.2, 0.6)
	Mean Positive Overshoot (%)	6.5	6.1	13.6	66.7
**Overall**	Headcount (%) (95%CI)	27.5 (26.0, 29.1)	16.0 (14.8, 17.3)	10.1 (9.1, 11.2)	7.9 (6.9, 8.8)
	Overshoot (%) (95%CI)	3.4 (2.3, 4.5)	2.5 (1.4, 3.6)	2.0 (0.9, 3.1)	1.7 (0.7, 2.8)
	Mean Positive Overshoot (%)	12.4	15.6	19.8	21.5

The intensity of catastrophe for the entire sample (i.e. the ‘overshoot’) at a 10% threshold was 2.5% i.e., on an average households spent 2.5% beyond the 10% catastrophic threshold. However, not all households actually experienced financial catastrophe. Households that actually experienced catastrophe at the 10% threshold spent an average of 15.6% beyond the threshold i.e., the ‘mean positive overshoot’. Thus, these households spent an average of 25.6% of their income on OOP payments i.e., threshold + mean positive overshoot. The poorest households suffered the greatest overshoot and the second greatest mean positive overshoot: the greatest being in the fourth quintile. The overshoot decreased with an increase in the catastrophic threshold value, while the reverse was observed for the mean positive overshoot (Table 3).

Low household income, a ‘below the poverty line’ household status, the use of referral and/or super-specialty hospitals as place for consultation, and smaller households (having four or fewer members) were associated with a greater likelihood of incurring financial catastrophe at all the catastrophic thresholds (Table 4).

**Table 4:** Correlates of financial catastrophe among households

Independent variables*	**Odds ratio (95%CI) for incurring financial catastrophe at different thresholds**			
	**5%**	**10%**	**15%**	**20%**
**Poorest/first quintile**	3.7 (2.8, 5)	3.7 (2.6, 5.3)	7.8 (4.5, 14.5)	47 (12.2, 397.2)
(Least poor/fifth quintile)	p<0.05	p<0.05	p<0.05	p<0.05**
**Below poverty line card holders**	1.5 (1.1, 2)	1.9 (1.4, 2.6)	2.3 (1.6, 3.3)	2.7 (1.8, 4)
(Above poverty line card holders)	p<0.05	p<0.05	p<0.05	p<0.05
Government sector***	0.9 (0.7, 1.1)	1 (0.8, 1.3)	1.2 (0.9, 1.7)	1.4 (1, 2)
(Private sector)	p=0.44	p=0.89	p=0.16	p=0.05
**Super-specialty hospitals**	1.9 (1.5, 2.4)	2.1 (1.6, 2.8)	2.3 (1.7, 3.2)	2.3 (1.6, 3.4)
(Clinics/Health centres)	p<0.05	p<0.05	p<0.05	p<0.05
**Referral hospitals**	1.6 (1.3, 1.9)	1.5 (1.2, 1.9)	1.6 (1.7, 3.2)	1.5 (1.1, 2.1)
(Clinics/Health centres)	p<0.05	p<0.05	p<0.05	p<0.05
**Households with four or less members**	1.5 (1.3, 1.8)	1.8 (1.5, 2.2)	2.2 (1.7, 2.8)	2.2 (1.7, 2.9)
(Households with more than four members)	p<0.05	p<0.05	p<0.05	p<0.05

*Comparator is provided in the bracket. **Fisher exact p value. ***173 households whose members exhibited mixed health seeking behavior (i.e. using government as well as private sector as place for consultation) were dropped from the analysis.

### Poverty-related measures

OOP payments for outpatient care pushed 0.9% of people with chronic conditions below the poverty line in KG Halli in a one-month period, nearly doubling the absolute number of people living in poverty (Table 5).

**Table 5:** Impact of OOP payments for outpatient care on poverty

**Measures of poverty**	**Gross of OOP payments (1)**	**Net of OOP payments (2)**	**Difference**	
			**Absolute (3=2-1)**	Relative (3/1)*100
**Poverty headcount ratio** (95%CI)	1% (0.7, 1.3)	1.8% (1.4, 2.2)	0.9%	91.6%
Standard error	0.002	0.002		
**Mean Poverty gap** (INR) (95%CI)	2.4 (1.48, 3.27)	19.5 (−5.26, 44.32)	17.2	724.1%
Standard error	0.457	12.643		
**Normalised poverty gap** (INR) (95%CI)	0.4% (0.246, 0.545)	3.3% (−0.876, 7.390)	2.9%	724.6%
Standard error	0.076	2.108		
**Mean positive poverty gap** (INR) (95%CI)	242.4 (191.8, 293.1)	1039.3 (−289.3, 2367.9)	796.9	328.7%
Standard error	24.963	666.160		
**Normalised mean positive poverty gap** (95%CI)	40.4% (31.99, 48.87)	173.3% (−48.24, 394.88)	132.9%	328.7%
Standard error	4.163	111.090		

The average extent by which individuals fell below the poverty line (i.e., mean poverty gap) also increased from INR 2.4 (USD 0.05) to INR 19.5 (USD 0.4) as a result of OOP payments. OOP payments further deepened the poverty by an average of INR 796.9 (USD 16.2) for those living below the poverty line i.e., mean positive poverty gap.

To cope with OOP payments, households borrowed money in 109 (4.2%) instances and occasionally sold and/or mortgaged their assets (See Additional file 3). Households from the lowest income quintile were significantly more likely to borrow money (OR=6.3, 95%CI=3-14.8) than the highest quintile. None of the households in the highest income quintile had to sell and/or mortgage their assets.

Households were significantly more likely to cope using their savings when the clinics/health centres were used as place for consultation compared with the referral hospitals and/or super-specialty hospitals (OR=1.6, 95%CI=1-2.5). Households using super-specialty hospitals as place for consultation had 2.3 times greater odds (95% CI=1.4-2.3) of borrowing money than the households using clinics/health centres. No significant difference was found in the use of coping mechanisms between the government and private sector.

## Discussion

### OOP payments and resultant financial catastrophe

In KG Halli, 69.6% of the households made OOP payments for outpatient care for chronic conditions. As a result, 16% of households suffered financial catastrophe at a 10% threshold. There are no other Indian studies with which to compare our findings. In fact, the incidence of catastrophe in KG Halli, resulting only from OOP payments for outpatient care for chronic conditions, is much greater than the incidence of catastrophe from OOP payments for overall healthcare (healthcare for all types of ailments) in Karnataka (9.9%) and is comparable to that in India (15.4%) [9]. Mondal et al. [11] revealed that in 2007, urban households in West Bengal spent 4.2% of their annual household expenditure on care for chronic conditions. This estimate is close to our estimate from KG Halli (i.e., 3.2%). In fact, our estimate is similar to that for the overall healthcare in urban Karnataka (3.3%) for the year 2004–2005 [21].

Thus, residents of KG Halli incur high OOP expenditure for outpatient care for chronic conditions. We also found that there was no significant difference in the incidence of OOP payments and financial catastrophe between the government sector, which is expected to provide free healthcare (or with nominal user fees), and the private sector as place for consultation. This finding seems to contrast with an earlier study that reported a greater likelihood of incurring financial catastrophe by households seeking healthcare from the private sector [22]. There may be several reasons for this finding.

First, 66.3% of the OOP payments were for medications during outpatient care for chronic conditions in KG Halli. High OOP spending on medications, even when government sector was used as place for consultation (66%), was likely due to the unavailability and/or frequent out-of-stock status of essential medications in the government sector. In KG Halli, where diabetes and hypertension are the most reported chronic conditions, one of the two government facilities does not stock anti-diabetic or anti-hypertensive medications, while they are frequently out of stock in the other facility. The median availability of generic medications, listed as the core medications by the World Health Organisation, at government facilities was only 12.5% in Karnataka [23]. Another major component of OOP payments for chronic conditions were expenses for laboratory investigations. In KG Halli, blood sugar testing is not conducted at either of the two government facilities. Thus, for medications and testing, the patients using government facilities in KG Halli as place for consultation must rely on private pharmacies and laboratories in the area, which leads to high OOP payments. Otherwise, patients must seek care from referral government hospitals/super-specialty hospitals, in which case they incur substantial travel expenditures. Our estimates indicate that 90.8% of OOP payments in the government sector come from expenditures for medications, laboratory testing, and travel.

High OOP spending for medications has remained a consistent feature in India and is not limited to chronic conditions. Estimates from the consecutive Consumer Expenditure Surveys (CESs) have revealed that in urban India, the greatest share of OOP spending has been on medications; 81.6% in 1993–94, 74.8% in 1999–2000, and 71.2% in 2004–2005 [9,24]. Segregated estimates available from the CES from the year 1999–2000 for urban India further suggests that the share of OOP payments on medications (69.6%) was more for outpatient care (56.3%) than inpatient care (13.3%) [24]. In general, the trade liberalisation and reforms for pharmaceutical policies (especially regarding price control) in the last decade have been argued to be responsible for making medications more expensive in India [9].

Second, there are user fees in government hospitals with subsidies/exemptions for people with below the poverty line ration cards. In KG Halli, only 10.5% of households have the below the poverty line ration cards. In fact, 39.2% of households in poor conditions do not possess the ration card, a document often needed to access subsidised healthcare. The remainder of households possess above the poverty line ration cards. Therefore, healthcare in government facilities is not entirely free for most of the sample population.

Finally, in KG Halli, the provision for outpatient care for chronic conditions is primarily by the private sector (nearly 22 clinics and 4 hospitals). Many of the private providers in KG Halli are informal providers (less/not qualified) and are likely to charge lower fees than qualified private providers. A study in Delhi slums also revealed that households were less likely to incur catastrophic expenditures when they sought care from informal/unregistered private providers rather than government providers, although this association was not statistically significant [25]. In essence, the government sector when used as place for consultation fails to provide affordable care to people with chronic conditions in KG Halli.

We found that the likelihood of incurring financial catastrophe was significantly greater when referral and/or super-specialty hospitals were used as place for consultation rather than clinics/health centres. This finding suggests that effective gate keeping with enhanced coordination across the levels of healthcare services may help to reduce financial catastrophe for patients with chronic conditions. We could not find other studies from India reporting the incidence of catastrophic expenditures by the levels of healthcare services sought.

We found that households with four or fewer members were more likely to incur financial catastrophe than larger families. With 67% of the population in the productive age of 15 to 60 years, it is reasonable to assume that larger households would have more earning members and more income, thereby making them less likely to face financial catastrophe. Our finding corroborates similar association found in earlier studies [11,25].

### OOP spending is inequitably high among the poor

We found that in KG Halli, the incidence of financial catastrophe is higher among poor compared with rich and that poor spends higher share of their income as OOP payments. This is reverse in case of overall healthcare (inpatient and outpatient) in Karnataka and at the India level [9,24]. However, if we examine the studies providing segregated information on OOP payments for outpatient care in urban India, the picture is different. These studies reveal that incidence of financial catastrophe and the OOP spending (as share of income) was higher among poor households [22,26]. A study from West Bengal also revealed that although OOP payments for inpatient care were progressive, they were regressive for outpatient care [12].

These findings raise the question of whether the catastrophic payments are regressive in the case of outpatient care, even when they appear to be progressive for healthcare on the whole. This is an important question, as people with chronic conditions are more likely to spend repeatedly and incur greater cumulative expenses for outpatient care. The West Bengal study revealed that the odds of incurring financial catastrophe was greatest for outpatient care for chronic conditions, greater than that for inpatient care at various catastrophic thresholds [11]. Apart from the incidence, even the intensity of CHE was greatest among the poorest families in KG Halli. When poor households spend a greater part of their income as OOP payments, the absolute disposable income left with these households would be very less compared to rich households resulting in extreme financial distress.

### OOP payments push people into poverty

In KG halli, OOP spending on outpatient care not only pushed people into poverty but also deepened the poverty they suffered. Berman and colleagues [10] revealed that in 2004, nearly 4.9% of Indians fell below the poverty line due to OOP payments for outpatient care. In fact, the proportion of Indians falling below the poverty line due to OOP spending on healthcare has increased over the last decade [9]. To cope with OOP payments, most households used their savings, but for some ailments, they had to borrow money (3.4%) or even sell/mortgage their assets (0.2%). The West Bengal study reported that 11.3% of households borrowed money, while 0.5% of households had to sell and/or mortgage their assets to cope with OOP payments for outpatient care [12].

### Need to decompose expenditure analyses for chronic conditions

Considering the rising burden of chronic conditions in India and the fairly predictable need for long-term outpatient care for such conditions, segregated data about healthcare spending and its implications for outpatient care would be very useful for health managers/planners. Indrani Gupta [21], in her paper on poverty estimation methods presented to the Planning Commission of India, also suggests the need to account for the differences in ‘acute vs. chronic conditions’ and ‘hospitalisations vs. outpatient care’ while measuring OOP payments and related poverty measures.

### Untreated chronic conditions

In our study, respondents reported cases where family members were advised to use daily long-term medication but were unable to take it for various reasons. Such cases amounted to 3.1% of all reported chronic conditions. Analyses of the NSS from 2004 with regard to cardiovascular diseases and diabetes revealed estimates of untreated ailments similar to those of our study i.e., 4% of cardiovascular and 3% of diabetes cases [27]. We did not attempt to understand the reasons for the lack of treatment, but it seems logical to assume that financial constraints would be one of the primary reasons. Selvaraj and Karan [8] report that financial constraints have remained the second major reason for not seeking healthcare in India for the last two decades, explaining 20% of non-treated ailments in urban India in 2004.

### Study limitations

Our interest in studying the financing of outpatient care for chronic conditions grew from our work in KG Halli, where chronic conditions are highly prevalent and people face difficulties in accessing healthcare services. This limited focus should be remembered while considering our findings. In fact, the total OOP payments by households for overall healthcare could be much greater, and some of the associations we explored in this paper would be affected by these additional OOP payments for other ailments, including for inpatient care.

Our operational definition of chronic conditions used in this study missed individuals with undiagnosed chronic conditions. Studies in India have shown validity of using self-reports of morbidity [28]. Furthermore, our analysis focuses on healthcare expenditure and so a possible underestimation of illness prevalence would not affect it. The use of household income instead of the consumption expenditure (or non-food expenditure) for the calculation of CHE may lead to the overestimation of the household’s capacity to pay and an underestimation of the true CHE incidence. Importantly, our approach ignores households that chose to forgo healthcare and thus do not make OOP payments on healthcare. In fact, such households are likely to suffer from greater opportunity costs and the direct impact of ill health. Although a long period (nine months) of data collection would have probably overcome the seasonal differences in healthcare spending, the cross-sectional data could only provide the transient effect of OOP payments. We could not capture the long-term effects of OOP payments on these families.

### The way forward

It is clear from our study that OOP payments on outpatient care for chronic conditions are causing significant impoverishment among people in KG Halli. Consistent with Samb and colleagues’ [2] argument, many of our study findings make a case for strengthening the existing healthcare system to improve access to quality care for chronic conditions. Our findings have direct implications for the resources for health systems (especially finances and medical products) and the way healthcare services are organised for delivering healthcare.

In the context of high OOP payments, it is important to provide financial protection for the population, thereby enabling people to access healthcare services. In context of very limited financial protection provided by government funded healthcare services in India, only approximately 25% of the population is covered by some form of health insurance [29]. Most of this limited coverage took place in the last few years, primarily through government-initiated health insurance schemes, and in particular, the Rastriya Swasthya Bima Yojana (RSBY), a national health insurance scheme that now covers approximately 100 million people living below the poverty line or working in the informal labour sector. However, except for a few federal government-initiated social insurance schemes that cover approximately 5% of the Indian population, these schemes do not cover outpatient care [29]. The Vajpayee Arogyasri, a health insurance scheme recently launched in Bangalore city that would cover residents of KG Halli, is also limited to inpatient surgical services [30].

Using the NSS data, Shahrawat and Rao [31] analysed the impact of various OOP payment scenarios (no payment for medication, no payment for inpatient care, no payment for outpatient care) on the incidence of catastrophic expenditures. They found that the maximum reduction in the incidence of catastrophic expenditures occurred when people did not have to pay for medications and/or outpatient care compared with a negligible reduction from subsidising the inpatient care. We join them in suggesting that schemes, such as RSBY, should increase the depth of coverage (or benefit package) to include medications and the breadth of coverage to include vulnerable families not necessarily falling below the poverty line as a way to significantly reduce OOP payment-related impoverishment. The recent launch of a pilot initiative to test the inclusion of limited outpatient care (consultations and medications) in RSBY is a welcome development [32].

On the health services front, improvements in the availability of medications and diagnostics within the underfunded government sector (especially at health centres) and the control of the costs of such services in the private sector are needed. In the pluralistic healthcare delivery system of KG Halli, the efforts made in the frame of the UHARP to improve coordination across the healthcare providers with an enhanced gate-keeping function at the primary care level could reduce the unnecessary financial burden on households and improve the care for chronic conditions.

We only discuss the healthcare payments related impoverishment in this paper. It is important to consider this in context of the adverse social determinants that affect health and living conditions of urban poor communities. Limited access to drinking water, sanitation facilities, and education adversely affect their health and productively leading to deprivation [33]. Also, like in many low- and middle-income countries, India exhibits a ‘mixed health systems syndrome’ of low public financing, an unregulated private market, and poor governance in the health sector requiring reforms within and outside of the health sector [34].

## Conclusions

Most households in KG Halli make OOP payments on outpatient care for chronic conditions, be it in the private or (underfunded) government sector. As a result, some of these families suffer financial catastrophe and slip into poverty. Most families use their savings to cope, but some have to borrow money and/or sell their assets to handle catastrophic OOP payments for healthcare. There is a need to provide financial protection to families, especially those from the poorer sections of society, to protect these families from the impoverishing effect of OOP spending on healthcare. Existing government-initiated health insurance schemes, such as RSBY, should include outpatient care for chronic conditions. We also suggest strengthening the existing healthcare services by enhancing the gate-keeping function of the primary care services and the availability of affordable medications and diagnostics for common chronic conditions.

## Competing interests

UB, BST, RD and CMM are involved in implementation of the Urban Health Action Research Project. The authors declare that they have no competing interests.

## Authors’ contributions

ND conceptualised the study (Census). RD and ND designed the questionnaire. RD, BST, CMM, and UB supervised the data collection and did data validation. UB and TBS, with inputs from PK, prepared the dataset for analysis. UB conceptualised the analytical approach used in this paper, analysed the data and wrote the draft manuscript. RD, ND, PK, and BC reviewed the commented on the manuscript. All the authors read and approved the final manuscript submitted to the journal.

## Pre-publication history

The pre-publication history for this paper can be accessed here:

http://www.biomedcentral.com/1471-2458/12/990/prepub

## Supplementary Material

Additional file 1 **Correlates of financial catastrophe among households.** Provides a table providing monthly OOP payments per chronic condition according to type and level of healthcare services. Click here for file

Additional file 2 **Concentration curves and indices.** Depicts the concentration curves for catastrophic healthcare expenditure at various catastrophic thresholds. It also provides values for concentration index for various catastrophic thresholds. Both, the concentration curve being above the line of equality, as well as the negative values for concentration index, suggest that the financial catastrophe is concentrated among the poor households. Click here for file

Additional file 3 **Source of finances for households to cope with OOP payments.** Provides a table presenting the data on source of financing used by households to cope with the out-of-pocket payments for outpatient care for chronic conditions. It provides this information for households in different income quintiles, households that fell above or below the poverty line, and according to levels of healthcare services used by households. Click here for file
