# Supplementary material for: Out-of-pocket healthcare payments on chronic conditions impoverish urban poor in Bangalore, India
Source: BMC Public Health. 2012 Nov 16;12:990. doi: 10.1186/1471-2458-12-990 (PMC3533578; doi:10.1186/1471-2458-12-990)
Supplement: Additional file 1 — Correlates of financial catastrophe among households. Provides a table providing monthly OOP payments per chronic condition according to type and level of healthcare services. [file 1471-2458-12-990-S1.pdf]

Table S1 Monthly OOP payments per chronic condition according to type and level of healthcare services

Type of the health Services as place for consultation	Level of the health services	OOP payments on outpatient care (in INR) Median (95% CL)		
		Total	Direct medical care	Others
**Government**	Clinics/ health centers	200 (150, 250)	200 (122.3, 250)	0 (0, 0)
	Referral hospitals	250 (200, 386.9)	150 (0, 300)	60 (50, 100)
	Super-specialty hospitals	280 (230, 300)	120 (0, 300)	50 (32, 60.6)
	Overall	250 (200, 300)	150 (100, 200)	32 (32, 50)
**Private**	Clinics/ health centers	300 (290, 340)	300 (270, 306.4)	0 (0, 0)
	Referral hospitals	480 (450, 500)	450 (410, 500)	0 (0, 0)
	Super-specialty hospitals	405 (330, 590)	375 (300, 550)	30 (30, 30)
	Overall	400 (370, 400)	360 (350, 400)	0 (0, 0)
