# Supplementary figures and images for: Out-of-pocket healthcare payments on chronic conditions impoverish urban poor in Bangalore, India
Source: BMC Public Health. 2012 Nov 16;12:990. doi: 10.1186/1471-2458-12-990 (PMC3533578; doi:10.1186/1471-2458-12-990)

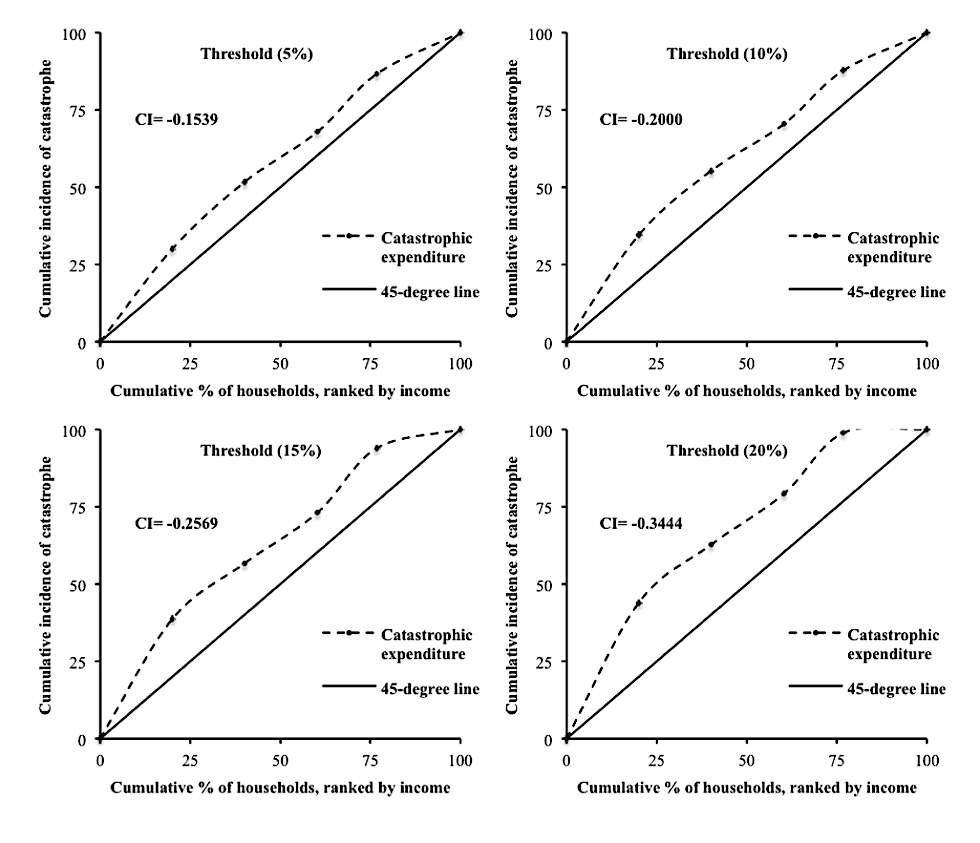

Supplement: Additional file 2 — Concentration curves and indices. Depicts the concentration curves for catastrophic healthcare expenditure at various catastrophic thresholds. It also provides values for concentration index for various catastrophic thresholds. Both, the concentration curve being above the line of equality, as well as the negative values for concentration index, suggest that the financial catastrophe is concentrated among the poor households. [file 1471-2458-12-990-S2.png]
