# Supplementary material for: Out-of-pocket healthcare payments on chronic conditions impoverish urban poor in Bangalore, India
Source: BMC Public Health. 2012 Nov 16;12:990. doi: 10.1186/1471-2458-12-990 (PMC3533578; doi:10.1186/1471-2458-12-990)
Supplement: Additional file 3 — Source of finances for households to cope with OOP payments. Provides a table presenting the data on source of financing used by households to cope with the out-of-pocket payments for outpatient care for chronic conditions. It provides this information for households in different income quintiles, households that fell above or below the poverty line, and according to levels of healthcare services used by households. [file 1471-2458-12-990-S3.pdf]

Table S2 Source of finances for households to cope with OOP payments

Independent variables		Percentage distribution of households according to source of finance [n (%)]*		
		From savings	From borrowing money	From selling or mortgaging of assets
**Income**	1st quintile	449 (91.5)	41 (8.4)	4 (0.8)
	2nd quintile	457 (96)	23 (4.8)	2 (0.4)
	3rd quintile	487 (97)	15 (3)	1 (0.2)
	4th quintile	426 (97)	14 (3.2)	1 (0.2)
	5th quintile	621 (98.9)	9 (1.4)	0 (0)
**Type of the health services as place for consultation**	Government	484 (94)	29 (5.6)	4 (0.8)
	Private	2001 (96.5)	80 (3.9)	5 (0.2)
**Levels of the health services**	Clinics/Health centers	1035 (97)	36 (3.4)	3 (0.3)
	Hospitals	972 (96.7)	35 (3.5)	4 (0.4)
	Super-specialty hospitals	478 (92.6)	38 (7.5)	2 (0.4)
**Overall**		2494 (96)	109 (4.2)	9 (0.4)

*Total may cross 100% due to multiple responses (sources of finance) by single household.
